# Indicators and determinants of the years of working life lost: a narrative review

**DOI:** 10.1177/1403494821993669

**Published:** 2021-03-01

**Authors:** Rahman Shiri, Aapo Hiilamo, Tea Lallukka

**Affiliations:** 1 Finnish Institute of Occupational Health, Helsinki, Finland; 2 Department of Public Health, University of Helsinki, Helsinki, Finland

**Keywords:** Chronic disease, working life expectancy, retirement, sick leave, work, social determinants of health, health behaviour

## Abstract

**Objective:**

This narrative review summarizes the available indicators for working life expectancy and years of working life lost (YWLL) and their determinants.

**Methods:**

We searched PubMed and Embase databases from their inception until August 2020 and screened all studies proposing an indicator for working life expectancy or YWLL. We also reviewed studies focusing on sociodemographic, lifestyle and work-related determinants of working life expectancy and YWLL. The results were synthesized narratively.

**Results:**

We identified 13 different indicators for the length of working life or YWLL. The most frequently used indicators were ‘working life expectancy’, ‘healthy working life expectancy’, and YWLL. Working life expectancy and healthy working life expectancy are longer for men than women. Working life expectancy at the age of 50 has been increasing since the mid-90s, and the increase has been larger for women, reducing the sex difference. Working life is shorter for people with a low level of education, in lower occupational classes, for people exposed to high physical work demands, those living in the most socioeconomically deprived areas, people with overweight or obesity, smokers, people who are inactive during leisure time and in people with a chronic health problem.

**Conclusions:**

**Despite increasing interest in understanding the determinants of YWLL, only a few studies have simultaneously considered multiple exit routes from the labour market. We propose a new measure for total YWLL considering all relevant exit routes from employment. This comprehensive measure can be used to assess the effect of given policy changes on prolonging working life.**

## Introduction

Due to population ageing, many countries have put forward an extensive set of policy measures to lengthen working life and to sustain work participation rates. These policy measures include increasing the statutory retirement age [1-4], limiting the availability of early voluntary retirement [2, 3], tightening eligibility for disability retirement [3, 5] and implementing targeted prevention programmes for work disability.

It is well-established that poor health is the most common barrier to extending working life [3, 6, 7]. People with a chronic disease are more likely to have more unstable work careers involving, for example, higher rates of unemployment and work disability in their early careers [8]. They spend more time on sick leave and disability retirement and retire earlier than people without chronic health problems [6, 9-11]. For example, of Canadian workers aged 50–62 without health problems, 55% remained in employment at the age of 64, whereas 33% of workers with activity limitations and a decline in general health were employed at the age of 64 [12]. Moreover, workers with health problems are less likely to work beyond retirement age than workers without health problems [13, 14]. In Sweden, people who continued working beyond the normal retirement age of 65 years reported on average 7% better self-rated health during retirement than people who retired at the age of 65 [15]. This advantage, however, disappeared 6 years after retirement and did not improve physical fitness, well-being or depressive symptoms [15].

Several different indicators to estimate the length of working life [4, 16] or the number of working years lost [17-19] have been proposed. However, most of the original studies identified the determinants of a single exit route from employment, such as sickness absence, disability retirement, early retirement, unemployment, non-employment or premature death. Only a few studies [17, 18] combined several of these exit routes and calculated summary measures, such as working life expectancy (WLE) or the number of working years lost, which provide policymakers with a more tangible and comprehensive picture regarding the length of working life and its determinants. For example, measures of the years of working life lost (YWLL) give more weight to death or disability retirement at younger ages [17, 20]. Individuals with mental disorders are generally granted disability retirement at younger ages [17, 21], thus, disability retirement due to mental disorders can cause a higher number of working years lost than disability retirement due to musculoskeletal disorders or any other somatic disorder [17, 21]. Appropriate measurements of length of working life and YWLL, and understanding their determinants are crucial for planning effective policy measures to extend working life.

The current narrative review had two aims. First, to comprehensively identify all the available indicators for length of working life and YWLL as well as their sociodemographic, lifestyle and work-related determinants. Second, if the current indicators could be improved, to propose a new indicator for YWLL, building upon current understanding and aiming to fill in potential gaps in the identified indicators.

## Methods

The first author systematically searched PubMed and Embase databases from their inception up to and including August 2020. MeSH terms and text words were used in PubMed, and a combination of Emtree terms and text words were used in Embase (Table I). The source of publications was limited to Embase (not Embase and Medline). We included all studies on any indicators of WLE or working years lost and did not exclude studies that proposed new indicators. We also screened the studies on sociodemographic, lifestyle and work-related determinants of WLE and YWLL. The first and the second authors screened the relevant papers for their applicability. We also reported some determinants of YWLL due to a single exit route from the labour market, such as disability retirement, unemployment or early retirement. However, the search strings were not developed for a single exit route from employment. The current review therefore did not include all relevant studies on YWLL due to a single exit rout. Moreover, we did not evaluate the methodological quality of the included studies. Quality assessment is not relevant for studies on indicators and there were only a limited number of studies on the determinants of WLE and YWLL due to more than two exit routes from employment.

**Table I.** PubMed and Embase searches made on 10 August 2020.

Search	PubMed		Embase only	
	Query	No. of items found	Query	No. of items found
#1	quality-adjusted life years[Mesh] OR life expectancy[Mesh] OR years lost[tiab]	29,634	‘quality adjusted life year’/exp OR ‘life expectancy’/exp OR ‘years lost’:ab,ti	64,646
#2	work[Mesh] OR work[tiab] OR working[tiab]	1,234,401	‘work’/exp OR working:ab,ti	434,511
#3	#1 AND #2	1484	#1 AND #2	2176
#4	life work[tiab] OR lost work[tiab] OR working life[tiab]	2757	‘life work’:ab,ti OR ‘lost work’:ab,ti OR ‘working life’:ab,ti	2727
Final	#3 OR #4	4181	#3 OR #4	4848

## Results

### Indicators

The search string retrieved 4181 publications in PubMed and 4848 in Embase. We identified the following 13 indicators for length of working life or number of working years lost. The commonly used indicators were WLE, healthy working life expectancy (HWLE), and YWLL.

#### Working life expectancy

WLE is defined as the number of years a person is expected to work over a lifetime [18, 22, 23]. Studies have analysed working life expectancies from two analytical perspectives: the cohort perspective, that is, the average lifetime spent in work of an actual birth cohort; and the period perspective, that is, the expectancy of a fictional cohort who would experience stable levels of work participation rates. Some studies defined WLE as the number of years a person is expected to be in paid employment, others as the number of years a person is economically active, including years spent in both employment and unemployment [18, 24].

In some official statistics the length of working life is defined as WLE at the age of 15 [24]. However, studies focusing on the older population have defined it, for example, as average time spent in employment between 50 and 74 [23]. WLE estimates vary substantially, depending on analytical approach (period vs. cohort perspective and Sullivan vs. multi-state modelling) [16, 22], country context, sex, and socioeconomic factors. For example, in Europe, from 1995 to 2001, average WLE at the age of 50 using the multi-state life table approach was 9.4 years for men and 6.4 years for women [16]. For men this ranged from 7.0 to 7.3 years in Austria, Belgium and France and from 10.9 to 11.4 years in Denmark, Greece, Portugal and the United Kingdom; for women, WLE was 3.3 years in Italy and ranged from 8.8 to 9.4 years in Denmark, Finland and Germany [16]. Only in Finland were women found to work a few months longer than men [16]. In 2009, WLE at the age of 50 for men using the Sullivan method ranged from 7.4 years in Hungary to 13.0–13.9 years in Norway, Sweden and Switzerland to 16.5 years in Iceland, and for women it ranged from 5.6 to 5.9 years in Italy, Luxembourg and Poland to 11.6–11.9 years in Denmark, Norway and Sweden to 13.6 years in Iceland [24]. In Estonia, Finland and Latvia, WLE at the age of 50 for men was a few months longer for women than men. The difference in WLE between men and women in 2009 was largest (3.7–4.2 years) in Ireland, Italy and Spain [24]. In Finland, using the Sullivan method, WLE at the age of 50 was 10 years for women and 9 years for men in 2012 [22]; it was 10 years for both men and women between 2005 and 2014 using the multi-state life table approach [25]. WLE at the age of 50 has been increasing across Europe and the United States since the mid-90s [22-24]. However, the increase has been larger in women than in men, leading to a decrease in the sex difference in WLE [24].

#### Healthy working life expectancy

Healthy WLE is defined as the number of years on average a person can expect to work in good health (without a limiting long-standing illness) between 50 and 70 years of age [4, 16]. Healthy WLE is higher for men than for women. In Europe, it ranges between 5.5 and 9.7 years for men and between 2.9 and 8.3 years for women [4, 16, 26, 27]. During 1995–2001, average healthy WLE at the age of 50 in Europe was 7.5 years for men and 4.8 years for women [16]. This ranged from 5.5 years in France to 9.7 years in Greece for men and from 2.9 years in Italy to 6.1–6.2 years in Denmark and Finland for women [16]. It was 9.4 years for men and 8.3 years for women in England during 2002–2013 [4].

#### Years of working life lost

YWLL is defined as a summary measure of different exits from the labour market [18]. However, the majority of previous studies have not used YWLL as a summary measure of different exits from employment; they determine YWLL to be a single premature exit route such as disability retirement, early retirement or unemployment. Only two studies included three [17] or several [18] exit routes from the labour market in their calculation of YWLL. Disability retirement is a common cause of YWLL [17-18].

### Other indicators

#### Occupationally active life expectancy

Occupationally active life expectancy measures the average age at which work capacity ends [28]. With no occurrence of disability pension or death before retirement, the occupationally active life expectancy would be equal to statutory retirement age [28].

#### Disability-adjusted working life years

Disability-adjusted working life years (DAWLYs) estimate the number of working years lost due to a disease or its consequences [29]. One DAWLY represents one lost year of heathy working life. The calculation of DAWLYs is similar to that of disability-adjusted life years [30]. DAWLYs for a disease are calculated as the sum of working years lost due to premature death and the number of working years lost due to disability.

#### Potential gains in life expectancy

By not considering competing risks, the years of potential life lost to premature death can be overestimated [25]. Potential gains in life expectancy, which does incorporate competing risks into the model, estimates the potential gains if a particular cause of deaths can be eliminated [31]. This indicator can also be used for working population or as potential gains in WLE.

#### Working life expectancy scale

The working life expectancy scale (WLE scale) uses a visual analogue scale to assess a worker’s expectation of maintaining their current job [32]. Workers are required to respond to a single statement: ‘I can keep working in this job for 5 more years’. The response scale ranges from 0 (never) to 100 (always) at intervals of 10, higher scores indicating a higher expectation of maintaining the current job [32].

#### Lost-work opportunity score

The lost-work opportunity score consists of unemployment, forced retirement and earlier than planned retirement [33]. It considers early retirement due to temporary lay-offs, company buy-outs, forced relocations, and so forth [33].

#### Potential of years of working life lost

Potential of years of working life lost (PYWLL) is estimated as YWLL plus expected losses occurring from the absence of return-to-work. It describes the difference between official and actual retirement ages [19].

#### Inactivity ratio

The inactivity ratio is calculated as YWLL or PYWLL divided by active age range (15–65 years) [19]. It gives the percentage of time in inactivity. The inactivity ratio was 16% for YWLL and 30% for PYWLL in patients with osteoarthritis [19], and 13% for YWLL and 25% for PYWLL in patients with rheumatic diseases in Portugal [34].

#### Work environment disability-adjusted life year

Work environment disability-adjusted life year (WE-DALY) assesses the number of fatal and non-fatal injuries and illnesses occurring in industry because of exposure to workplace hazards [35]. It is the sum of the number of years of life lost by premature mortality in the worker population and the number of years of life lived with a disability for each non-fatal injury or illness [35].

#### The index of potential years of work tenure lost

The index of potential years of work tenure lost (IPYWL) is calculated by dividing the sum of potential years of work tenure lost (PYWL) (sum of the number of deaths in a working age group × remaining working years of that age group) by expected PYWL (PYWL × standard proportion [calculated by numbers of death in a working age group divided by all workers]) [36].

#### The work lost rate

The work lost rate is a measure of absenteeism [37]. It is calculated as ‘hours unpaid/(work hours paid + hours unpaid) × 100’. Workers younger than 30 years old had a higher work lost rate, while workers older than 60 years old had a lower work lost rate due to a small number of workers with longer absences [37]. Unpaid hours included sick days and absent days due to personal reasons.

### Determinants

We identified 18 original studies on sociodemographic, lifestyle and work-related determinants of WLE or YWLL due to two or more exit routes from the labour market. The current review also included studies on single exit routes from employment, particularly disability retirement, however the review of the determinants of single exit routes is not comprehensive.

#### Sociodemographic factors

Education was positively associated with labour force participation and WLE [24]. People with higher levels of education had a higher WLE than people with lower levels of education [18, 38]. In the Netherlands, low-educated men had a 7-year and less educated women had a 10-year lower WLE at the age of 30 than highly educated people [18]. This difference was 2.5 years for men and 3.4 years for women at the age of 50 [18]. In the Netherlands and the United States, educational inequalities in WLE are larger for men than for women [18, 38].

Healthy WLE was also inversely associated with level of education, which was longest for people with a tertiary education (11.3 years) in England [4].

Older workers (50 years+) with a low level of education more frequently exit from paid employment due to ill health than workers with a high level of education [39]. In the Netherlands, low-educated men lost 6.3 years due to unemployment and 3.4 years due to disability retirement, while highly educated men lost 1.9 and 0.8 years, respectively [18]. Low-educated women lost 7.0 years due to unemployment and 3.0 years due to disability retirement, whereas highly educated women lost 1.8 and 1.4 years, respectively [18]. Low-educated workers more commonly suffer from diabetes, cardiovascular diseases and musculoskeletal disorders than highly educated workers [40], and they lose more working years due to disability retirement than highly educated people [40, 41], particularly disability retirement due to musculoskeletal diseases [41].

Older workers (50 years+) with a low occupational grade more frequently exit from paid employment due to ill health than workers of a high occupational grade [39]. In Sweden, blue-collar workers lost more working years than white-collar workers [5]. In South Korea, manual workers were at increased risk of disability retirement [42]. In Finland, WLE at the age of 50 was 10.5 years for upper-level non-manual workers and 9.5 years for manual workers [25]. Self-employed Finnish workers had the largest WLE at the age of 50 [25], and in 2012, upper non-manual workers had a 3.5-year higher WLE than manual workers [22]. Healthy WLE (11.9 years) [27] and occupationally active life expectancy (62 years) [28] were highest for Finnish male executives, while occupationally active life expectancy was lowest for unskilled workers (52 years) [28]. In England, healthy WLE was longest for self-employed (11.8 years) and non-manual occupations (10.3 years) and was shortest for people with manual occupations (8.7 years) [4].

The 2008–2009 financial crisis reduced WLE [38, 43]. In Spain, the recession reduced WLE in men by 12 years and in women by 7 years [43]. WLE changed more among manual and unskilled workers than among non-manual and skilled workers [43]. The loss of working years was mainly due to unemployment or being out of the labour market (inactivity) [43]. In people aged 50, WLE declined by 1.5 years in men and by 0.5 years in women in the United States [38]. In a study conducted among Finnish men [28], marital status was associated with occupationally active life expectancy. Mean occupationally active life expectancy was shortest for single men (50 years) and longest for ever-married men (59 years).

In the United States, foreign-born people had a lower WLE at the age of 50 than native-born people [23]. The gap in the duration of working life at the age of 50 between native- and foreign-born people in the United States has increased over time [23]. Healthy WLE was inversely associated with area-level deprivation, and in England healthy WLE was longest for people living in the least deprived areas (10.5 years) and shortest for those in the most deprived area (6.8 years) [4].

#### Lifestyle risk factors

In South Korea, workers who did not exercise regularly or were underweight or overweight were more likely to retire before the age of 65 than workers who exercised regularly or were normal weight [44]. People who were physically active for at least 2 days a week were more likely to work beyond retirement age [45]. Disability retirement is more common in people with overweight or obesity [46], in smokers [42, 47] and in those who are inactive during their leisure time [48] compared with normal weight, never smokers or active individuals, respectively.

#### Work-related risk factors

In Denmark, at the age of 30, men exposed to high physical work demands had 2 years lower WLE than low-exposed men, and women exposed to high physical work demands had 3 years lower WLE than low-exposed women [49]. Workers exposed to high physical work demands lost more working years due to unemployment, sickness absence and disability retirement than low-exposed workers [25, 49]. In the Netherlands, workers who felt underappreciated at work or had a low focus on their developing skills and knowledge were more likely to retire early [50]. In older workers (50 years+) in England, psychosocial demands predicted preference for early retirement, while decision authority predicted preference for late retirement and reduced work exits [51]. Manual workers were at increased risk of disability retirement [42], and physically heavy jobs [52] or physical workload [25, 53] increased the number of working years lost due to disability retirement. The lifting or carrying of heavy loads, hand tool vibrations and hard physical work during working life increased the number of working years lost due to long-term sickness absence or disability retirement in Danish older workers [54].

In workers with a chronic disease, low supervisor and co-worker social support, and burnout predicted exits from paid employment [55]. In workers aged 45–63 with a chronic disease, a favourable change in physical workload reduced the rate of exit from paid employment [56].

#### Chronic diseases

Workers with a chronic disease exit paid employment due to disability retirement, unemployment and early retirement more frequently than workers without such conditions [55]. Cardiovascular disease, chronic mental illness, diabetes and chronic musculoskeletal disease are among the most frequent causes [55]. People with depressive symptoms spend more time unemployed and absent through sickness than those without depressive symptoms, particularly women [57]. In patients with osteoarthritis, early retirement contributed the most to YWLL, followed by unemployment and disability retirement [19]. Older Canadian workers suffering from mental- or musculoskeletal disorders were at increased risk of non-employment, while older workers suffering from diabetes or cardiovascular disease were at increased risk of early retirement [12]. A South Korean study reported an average reduction in WLE of 9.7 years because of disability retirement [42]. In Finland, impaired glucose metabolism reduced participation in working life [58], and disabling shoulder lesions reduced working life by 2 to 8 years compared with the general population [59]. In Canada, arthritis or rheumatism at the age of 15 were found to reduce WLE of men by 4 years and of women by 3 years [60].

Evidence from Northern European countries shows that disability retirement is awarded at a younger age for a mental disorder than for a musculoskeletal disorder or any other somatic disorder [21]. Thus, mental disorders cause more YWLL due to disability retirement than musculoskeletal disorders [21]. In Norway, depression and anxiety are the most common causes of working years lost due to mental disorders [21]. In Sweden, disability retirement due to psychiatric disorders was highest in men aged 20–29 and lowest in men aged 60–64 [52]. In the Netherlands, workers with poor physical health and those in a financial position to be able to stop working before statutory retirement age were more likely to retire early [50]. In workers with a chronic disease, both poor self-rated health and extent of activity limitation from the disease predicted exit from paid employment [55]. The number of pain sites and the severity of pain were associated with lower scores on the WLE scale [32].

### Summary of the results

Several studies have provided population estimates of WLE and healthy WLE. The estimates varied substantially because of differences in the analytical approaches and perspectives used, country contexts, periods, and demographic- and socioeconomic factors. Nevertheless, four noteworthy patterns emerged. First, individuals at the age of 50 can expect roughly 10 more years of employment. Second, at the age of 50, men tend to work longer than women. However, the increase in WLE has been larger for women than for men. Even in Estonia, Finland and Latvia, women’s WLE at the age of 50 surpassed that of men. Third, individuals in low socioeconomic positions have lower WLE than those in high socioeconomic positions. Educational inequalities in WLE have been larger for men than for women. Fourth, work-related factors such as high physical work demands, unhealthy lifestyle habits, and health problems including mental disorders, musculoskeletal disorders, diabetes, and cardiovascular diseases, substantially decrease working life.

## Discussion

In the present study we aimed to provide a fair and comprehensive overview of the available indicators of length of working life and YWLL and their sociodemographic, lifestyle and work-related determinants, and to propose potential directions for new research. Our search of the literature identified 13 indicators for monitoring the length of working life and its determinants. Building upon the identified indicators, we suggest the following comprehensive indicator for total YWLL to consider all relevant exit routes from the labour market:

$$\begin{array}{l} YWLL=\sum \left([\frac{\mathrm{sa}\leq 5}{250}+\frac{sa>5}{365.2}+\;ue_{g}+\;dr_{g}^{t}]\;⁄\;P_{g}\right)\\ \;\;\;\;\;\;\;\;+(\left[sra-ago_{g}\right]\times \left[\frac{\mathrm{erg}+\mathrm{drpg}+\mathrm{dg}}{\mathrm{Pg}}\right])\times 10000 \end{array}$$

where sa ⩽5 = sickness absence for 5 days or shorter of age-, sex- and occupational class-group; sa >5 = sickness absence longer than 5 days of age-, sex- and occupational class-group; ue_g_ = mid number of years of unemployment or out of the labour market of age-, sex- and occupational class-group;. dr^t^_g_ = mid number of years of temporary disability retirement of age-, sex- and occupational class-group; *P*_g_ = population of age-, sex- and occupational class-group; sra = mid country- and year-specific statutory retirement age of sex- and occupational class-group; ago_g_ = mid age of age-, sex- and occupational class-group; er_g_= mid early retirement years of age-, sex- and occupational class-group; dr^p^_g_ = mid number of years of permanent disability retirement of age-, sex- and occupational class-group; and d_g_ = mid number of years lost because of death between the age of 18 and statutory retirement age in age-, sex- and occupational class-group.

Since there are 250 working days in a year [35], we divided sickness absence of 5 days or shorter by 250 days and sickness absence longer than 5 days by 365.2 days, because calculations of sickness absences of 5 days or fewer do not usually include weekends. As additional education advances careers and enhances skills, unemployment did not include years of education.

Poor health is the most significant contributor to exits from the labour market and a common barrier to extending working life. People with a chronic disease less frequently participate in the labour force than people without health problems, particularly low-educated people [7]. Workers with a chronic disease at the age of 55 extended their working lives only by about 1.6 years between 1992 and 1996 and by 5.2 to 6.8 years between 2012 and 2016 [1]. Among leavers with a health problem, a transition into non-employment is more common, while among healthy leavers, a transition into early retirement is more common [12]. To date, only a few studies have identified the relative contribution of different illnesses to YWLL by focusing on age at exit from employment. The studies indicate that mental health disorders contribute substantially to YWLL.

The findings of this review indicate that excess body mass, smoking, lack of leisure-time physical activity and exposure to high physical work demands increase the risk of exit from employment. To lengthen working life, interventions to prevent premature deterioration of work ability should be started in young adulthood and midlife [26]. Worksite health promotion and career development interventions can help extend working life [3]. Favourable improvements in physical workload factors can reduce the rate of exit from paid employment in workers with a chronic disease [56], and physical activity at least twice a week during leisure time can extend working life [45].

To date, a comprehensive indicator for measuring YWLL has not been available. The results of the review suggest that YWLL should include all exit routes from labour market participation including unemployment, sickness absence, disability retirement, involuntary (forced) retirement, unplanned, early old-age retirement as well as premature death. Only two studies considered multiple exit routes from work participation in the calculation of YWLL [17, 18]. One study considered unemployment, disability retirement, early retirement, no income, and premature death as well as time spent in education, and emigration, but did not compute an overall YWLL [18]. The other study considered only mortality, sickness absence and disability retirement in their estimation of YWLL [17]. The key limitations of the existing indicators include lack of consideration of some exit routes such as short-term sickness absence, early retirement, or premature death. We have proposed a new comprehensive indicator for measuring YWLL, which could be used to assess the effects of workplace interventions and risk factors or to compare different nations, cities, workplaces, and time trends. The comprehensive nature of the proposed measure for total YWLL is not a limitation. In the absence of extensive data on different exit routes from employment, the measure can still be used with data on at least two exit routes from the labour market.

## Conclusions

WLE, healthy WLE, and YWLL are commonly used indicators for monitoring the length of working life. Low socioeconomic position, high physical work demands, unhealthy lifestyle habits, and health problems are linked to a decreased length of working life. Gender-related differences in WLE have been decreasing. However, educational inequalities related to WLE remain larger for men than for women. We have proposed a new indicator for YWLL that considers short- and long-term sickness absences, temporary and permanent disability retirement, involuntary and voluntary early retirement, unemployment and being out of the labour market, and premature death. This comprehensive measure can be used to assess the effects of given policy changes on prolonging working life.
